# Workshop: how to start the rest of your career as a psychiatrist: a few words with those who made it

**DOI:** 10.1192/j.eurpsy.2026.10262

**Published:** 2026-07-31

**Authors:** F. H. Kraxner

**Affiliations:** Forensic psychiatry, Psychiatric Services of Kanton Aargau (PDAG), Brugg/Windisch, Switzerland

## Abstract

**Abstract:**

Early career decisions in psychiatry shape not only individual professional trajectories but also the future quality, accessibility, and scope of mental health care. This workshop contribution reflects on key lessons from established psychiatric careers, focusing on how young psychiatrists can navigate the transition from training to long-term professional fulfillment while responding to evolving clinical, societal, and global challenges.

Drawing on personal experience and collective insights from senior colleagues, the talk addresses core themes such as finding a professional identity, balancing clinical excellence with personal well-being, integrating psychotherapy, research, leadership, and innovation, and remaining adaptable in a rapidly changing mental health landscape. Emphasis is placed on maintaining patient-centered care while expanding horizons through interdisciplinary work, global perspectives, and emerging technologies.

By sharing practical guidance, reflective insights, and realistic narratives of success and challenge, this contribution aims to empower early career psychiatrists to shape meaningful, sustainable careers that contribute to improving mental health care for individuals and societies alike.

**Disclosure of Interest:**

None Declared

